# Chicken bromodomain-containing protein 2 interacts with the Newcastle disease virus matrix protein and promotes viral replication

**DOI:** 10.1186/s13567-020-00846-1

**Published:** 2020-09-22

**Authors:** Zhiqiang Duan, Yifan Han, Lei Zhou, Chao Yuan, Yanbi Wang, Caiqin Zhao, Hong Tang, Jiaqi Chen

**Affiliations:** 1 Key Laboratory of Animal Genetics, Breeding and Reproduction in the Plateau Mountainous Region, Ministry of Education, Guizhou University, Guiyang, China; 2 College of Animal Science, Guizhou University, Jiaxiu South Road, Huaxi District, Guiyang, 550025 Guizhou China

**Keywords:** Newcastle disease virus, matrix protein, bromodomain-containing protein 2, viral replication

## Abstract

Bromodomain-containing protein 2 (BRD2) is a nucleus-localized serine-threonine kinase that plays pivotal roles in the transcriptional control of diverse genes. In our previous study, the chicken BRD2 (chBRD2) protein was found to interact with the Newcastle disease virus (NDV) matrix (M) protein using a yeast two-hybrid screening system, but the role of the chBRD2 protein in the replication of NDV remains unclear. In this study, we first confirmed the interaction between the M protein and chBRD2 protein using fluorescence co-localization, co-immunoprecipitation and pull-down assays. Intracellular binding studies indicated that the C-terminus (aa 264–313) of the M protein and the extra-terminal (ET) domain (aa 619–683) of the chBRD2 protein were responsible for interactions with each other. Interestingly, although two amino acids (T621 and S649) found in the chBRD2/ET domain were different from those in the human BRD2/ET domain and in that of other mammals, they did not disrupt the BRD2-M interaction or the chBRD2-M interaction. In addition, we found that the transcription of the *chBRD2* gene was obviously decreased in both NDV-infected cells and pEGFP-M-transfected cells in a dose-dependent manner. Moreover, small interfering RNA-mediated knockdown of chBRD2 or overexpression of chBRD2 remarkably enhanced or reduced NDV replication by upregulating or downregulating viral RNA synthesis and transcription, respectively. Overall, we demonstrate for the first time that the interaction of the M protein with the chBRD2 protein in the nucleus promotes NDV replication by downregulating chBRD2 expression and facilitating viral RNA synthesis and transcription. These results will provide further insight into the biological functions of the M protein in the replication of NDV.

## Introduction

Newcastle disease (ND) is an important avian infectious viral disease that causes neurological, respiratory, and gastrointestinal symptoms in poultry and may lead to devastating losses in the poultry industry worldwide [1]. The causative agent of ND is Newcastle disease virus (NDV), also known as avian paramyxovirus type 1, which belongs to the genus *Orthoavulavirus* of the subfamily *Paramyxoviridae* [2]. The genome of NDV is a non-segmented, negative-sense, single-stranded RNA that encodes eight proteins, including six structural proteins [the nucleocapsid protein (NP), phosphoprotein protein (P), matrix protein (M), fusion protein (F), haemagglutinin-neuraminidase protein (HN) and large polymerase protein (L)] as well as two non-structural proteins (V and W) derived from RNA editing of the P gene [3, 4]. Of all these viral structural proteins, the M protein is surrounded by the inner surface of the viral envelope and forms an outer protein shell around the nucleocapsid, which constitutes the bridge between the viral envelope and the nucleocapsid [5]. Like the M protein of most paramyxoviruses, the NDV M protein is also a multifunctional nucleocytoplasmic trafficking protein [6]. In addition to participating in assembly in the cytoplasm and the budding of progeny virions at the cell membrane later in infection [7], the NDV M protein is localized in the nucleus and nucleolus early in infection [8, 9], which may inhibit host cell transcription and protein synthesis [10]. Recent studies have shown that nuclear-cytoplasmic trafficking of the NDV M protein is mediated by its intrinsic nuclear localization signal (NLS) and nuclear export signals (NESs) [11, 12]. Additionally, M/NLS mutation results in a pathotype change of NDV and attenuates viral replication and pathogenicity [11], while M/NES mutation causes ineffective rescue of NDV [12], demonstrating that nucleocytoplasmic trafficking of the NDV M protein plays crucial roles in the virus life cycle.

In recent years, an increasing number of studies have focused on mutation of some amino acids in the NDV M protein, such as the N-terminal ^23^FPIV^26^ motif [13], basic amino acids R36 [14] and R42 [15], and C-terminal G275 and P276 [16], to understand its functions. Additionally, exploration of the interaction of the M protein with cellular proteins has also been used to study the roles of the M protein in the replication and pathogenesis of NDV. For example, the NDV M protein interacting with the human Bax protein is beneficial to elucidate the pro-apoptotic ability of NDV [17]. In addition, host charged multivesicular body protein 4B or nucleophosmin interacts with the NDV M protein, which is essential for the replication of NDV and the nucleolar targeting of the M protein, respectively [18, 19]. Importantly, a recent study found that the interaction of the NDV M protein with the antiviral protein viperin reduces virus replication, suggesting for the first time that the M protein is involved in NDV immune evasion [20]. In our recent studies, several cellular proteins were found to interact with the NDV M protein using a yeast two-hybrid screening system, of which the chicken transcriptional regulatory factor bromodomain-containing protein 2 (BRD2) was a novel M-interacting partner that may regulate the replication of NDV [11], but the precise function of this interaction in NDV replication remains unclear.

BRD2 belongs to the bromodomain and extra-terminal domain (BET) family and contains two tandem bromodomains (BD1 and BD2) and an extra-terminal (ET) domain, of which the bromodomain is required for the epigenetic regulation of gene transcription by BET proteins through interacting with nucleosomes within chromatin, and the ET domain fulfils its regulatory functions by recruiting specific effector proteins [21, 22]. BRD2 is reported to be a nuclear transcription factor kinase and acts as a transcriptional regulator with switch mating type/sucrose non-fermenting (SWI/SNF)-like functions that regulate chromatin remodelling, inflammatory responses, cell-cycle progression and so on [23–25]. More importantly, BRD2 can also bind transcriptional activators, such as E2F proteins, and co-activators, including TATA-binding protein (TBP)-associated factors (TAFs), histone acetyltransferases and histone deacetylases, to regulate the transcription of diverse genes [26]. The NDV M protein is localized in the nucleus early in infection and does not require other NDV proteins [8, 9]. However, which cellular proteins in the nucleus the NDV M protein interacts with and how these interactions regulate virus replication remain unknown. In this study, the role of chicken BRD2 (chBRD2) in the life cycle of NDV was investigated. We demonstrated for the first time that the NDV M protein directly interacts with the chBRD2 protein in the nucleus, which downregulates the expression of chBRD2 and facilitates viral RNA synthesis and transcription to promote NDV replication.

## Materials and methods

### Cells, viruses and antibodies

Chicken embryonic fibroblasts (DF-1) and human embryonic kidney 293T (HEK-293T) cells were purchased from the Cell Resource Center of Shanghai Institutes for Biological Sciences of the Chinese Academy of Sciences. The NDV strain expressing green fluorescent protein (GFP) (rSS1GFP) was generated in our previous study [11]. The virus was plaque purified three times in DF-1 cells and propagated once in specific pathogen-free (SPF) embryonated chicken eggs. Primary mouse anti-HA tag monoclonal antibody (T0008), mouse anti-Myc tag monoclonal antibody (T0001), rabbit anti-Myc tag polyclonal antibody (T0052), mouse anti-His tag monoclonal antibody (T0009), mouse anti-GST tag monoclonal antibody (T0007), mouse anti-GFP tag monoclonal antibody (T0005), mouse anti-GAPDH monoclonal antibody (T0004), Alexa Fluor 488-conjugated goat anti-mouse IgG (H + L) antibody (S0017) and Cy3-conjugated goat anti-rabbit IgG (H + L) antibody (S0011) were purchased from Affinity Biosciences (USA). Rabbit anti-NDV M polyclonal antibody was prepared by Wuhan GeneCreate Biological Engineering Co., Ltd (China).

### Plasmid construction

The plasmids pEGFP-C1, pDsRed-C1, pCMV-HA, and pCMV-Myc were purchased from Clontech (USA), and pET-32a(+) and pGEX-6p-1 were purchased from Novagen (USA) and GE Healthcare (USA), respectively. All enzymes used for cloning procedures were purchased from Thermo Fisher Scientific Company (USA). The open reading frame (ORF) of the *M* gene (GenBank No. KP742770) was amplified from the plasmid pNDV/SS1GFP [11] and then subcloned into the plasmids pEGFP-C1, pCMV-HA, and pGEX-6p-1 to generate pEGFP-C1-M, pCMV-HA-M and pGEX-6p-M, respectively. The ORF of the *chBRD2* gene (GenBank No. XM_015294960) was amplified from cDNA derived from DF-1 cells and then used to construct the recombinant expression plasmids pDsRed-chBRD2, pCMV-Myc-chBRD2, and pET-32a-chBRD2. Plasmids expressing the truncation mutants or deletion mutants of the M and chBRD2 proteins were constructed by inserting PCR-generated fragments into the plasmid pEGFP-C1, pCMV-HA or pCMV-Myc. The gene fragments of chBRD2/ET and human BRD2/ET (huBRD2/ET) (GenBank No. NM_005104) were amplified from cDNA derived from DF-1 cells and HEK-293T cells, respectively, and then subcloned into the plasmid pCMV-Myc to generate pCMV-Myc-chBRD2/ET and pCMV-Myc-huBRD2/ET. Primers used for the construction of the above recombinant expression plasmids are shown in Table 1. All of the constructed recombinant expression plasmids were confirmed by PCR, restriction digestion and DNA sequencing.

**Table 1:** Primers used for the construction of recombinant expression plasmids

Recombinant plasmid	Sense primer (5′ → 3′)	Anti-sense primer (5′ → 3′)	Restriction sites^a^
pEGFP-M	TCG*GAATTC*AATGGACTCATCCAGGAC	GCA*GTCGAC*TTATTTCCTGAAAGG	EcoRI/SalI
pCMV-HA-M	TT*GAATTC*GGATGGACTCATCCAGGAC	GCA*CTCGAG*TTATTTCCTGAAAGG	EcoRI/XhoI
pGEX-6p-M	TGC*GAATTC*ATGGACTCATCCAGGAC	GGCA*CTCGAG*TTATTTCCTGAAAGG	EcoRI/XhoI
pDsRed-chBRD2	CAG*CTCGAG*CTATGCTGCAGAATGTGAATCC	TG*GGATCC*TTAACCCGAATCCGAGTCGCT	XhoI/BamHI
pCMV-Myc-chBRD2	AG*GAATTC*AAATGCTGCAGAATGTGAATCC	TG*CTCGAG*TTAACCCGAATCCGAGTCGC	EcoRI/*Xho*I
pET-32a-chBRD2	CAG*GAATTC*ATGCTGCAGAATGTGAATCC	TG*CTCGAG*TTAACCCGAATCCGAGTCGC	EcoRI/XhoI
pCMV-HA-M(1–178)	TT*GAATTC*GGATGGACTCATCCAGGAC	GA*CTCGAG*TTACAAGGAGACAAAATTCAC	EcoRI/XhoI
pCMV-HA-M(1–245)	TT*GAATTC*GGATGGACTCATCCAGGAC	AA*CTCGAG*TTATACAGTGGACATAAGCCC	EcoRI/XhoI
pCMV-HA-M(1–263)	TT*GAATTC*GGATGGACTCATCCAGGAC	GA*CTCGAG*TTATCTCCTTATCTTTTCCTC	EcoRI/XhoI
pCMV-HA-M(264–364)	GA*GAATTC*GGCTCAATCTATCTGTTGG	GCA*CTCGAG*TTATTTCCTGAAAGG	EcoRI/XhoI
pCMV-HA-M(314–364)	GA*GAATTC*GGATACTCTGGAGCCAGACC	GCA*CTCGAG*TTATTTCCTGAAAGG	EcoRI/XhoI
pCMV-HA-M(264–313)	GA*GAATTC*GGCTCAATCTATCTGTTGGG	CT*CTCGAG*TTACTTGGCAACCTGGGGAGA	EcoRI/XhoI
pCMV-Myc-chBRD2(1–179)	AG*GAATTC*AAATGCTGCAGAATGTGAATCC	TC*CTCGAG*TTACATTTGGGCCACCTTCTGCA	EcoRI/XhoI
pCMV-Myc-chBRD2(1–447)	AG*GAATTC*AAATGCTGCAGAATGTGAATCC	GG*CTCGAG*TTAGTAGCTGAACTCAAAGACATCC	EcoRI/XhoI
pCMV-Myc-chBRD2(1–510)	AG*GAATTC*AAATGCTGCAGAATGTGAATCC	TC*CTCGAG*TTAGTCAGAGCTCTCCTCGCTCTCT	EcoRI/XhoI
pCMV-Myc-chBRD2(511–779)	GA*GAATTC*TCTCGGAAGAGGAACGTGCCAACC	TG*CTCGAG*TTAACCCGAATCCGAGTCGC	EcoRI/XhoI
pCMV-Myc-chBRD2(619–683)	AG*GAATTC*AACCGATGACGTACGATGAGAAG	TT*CTCGAG*TTAGGACAGCACGTAGCGCTCCAG	EcoRI/XhoI
pCMV-Myc-chBRD2/ET	AG*GAATTC*AACCGATGACGTACGATGAGAAG	TT*CTCGAG*TTAGGACAGCACGTAGCGCTCCAG	EcoRI/XhoI
pCMV-Myc-huBRD2/ET	AG*GAATTC*AACCCATGAGTTACGATGAGAAG	TT*CTCGAG*TTAGGACAGCACGTAGCGCTCCAG	EcoRI/XhoI
pCMV-HA-M(Δ264-313)	TT*GAATTC*GGATGGACTCATCCAGGAC	CTGGCTCCAGAGTATTCTCCTTATCTTTTCCTC	*Eco*RI
	GAAAAGATAAGGAGAATACTCTGGAGCCAGACC	GCA*CTCGAG*TTATTTCCTGAAAGG	*Xho*I
pEGFP-M(Δ264-313)	TCG*GAATTC*AATGGACTCATCCAGGAC	CTGGCTCCAGAGTATTCTCCTTATCTTTTCCTC	*Eco*RI
	GAAAAGATAAGGAGAATACTCTGGAGCCAGACC	GCA*GTCGAC*TTATTTCCTGAAAGG	*Sal*I
pCMV-Myc-chBRD2(Δ619-683)	AG*GAATTC*AAATGCTGCAGAATGTGAATCC	TTTCTTCCGCAGGCATTTGCTCTCCTCTTCCTC	*Eco*RI
	GAAGAGGAGAGCAAATGCCTGCGGAAGAAACCC	TG*CTCGAG*TTAACCCGAATCCGAGTCGC	*Xho*I

^a^Restriction sites are given in italics and underlined.

### Cell culture, transfection and fluorescence microscopy

DF-1 cells were maintained in Dulbecco’s modified Eagle medium (Gibco, USA) containing 12% foetal bovine serum (FBS) (Gibco, USA) at 37 °C under an atmosphere with 5% CO_2_. For the transfection experiments, 5 × 10^5^ DF-1 cells were grown to 80% confluence in 6-well plates and then double (pEGFP-M and pDsRed-chBRD2 or pCMV-HA-M and pCMV-Myc-chBRD2) transfected with a total of 3 μg of plasmid (1.5 μg for each plasmid) using FuGENE HD Transfection Reagent (Promega, USA) according to the manufacturer’s instructions. Twenty-four hours after transfection, DF-1 cells expressing the recombinant proteins EGFP-M and DsRed-chBRD2 were rinsed with phosphate-buffered saline (PBS), fixed with pre-cooled 4% paraformaldehyde for 20 min at room temperature, permeabilized with 0.25% Triton X-100 for 5 min, and then counterstained with DAPI (Sigma, USA) to detect nuclei. For the fluorescence detection of the recombinant proteins HA-M and Myc-chBRD2, plasmid-co-transfected DF-1 cells were rinsed, fixed, permeabilized as described above, blocked with 10% FBS in PBS for 1 h, and then incubated with mouse anti-HA and rabbit anti-Myc antibodies for 1 h. After three washes with PBS, the cells were incubated with Alexa Fluor 488-conjugated goat anti-mouse IgG (H + L) and Cy3-conjugated goat anti-rabbit IgG (H + L) antibodies for 1 h. Cells were also counterstained with DAPI to detect nuclei. Fluorescent images were obtained under an inverted fluorescence microscope and then analysed and merged with Adobe Photoshop CS7 software.

### Protein interaction assays

For co-immunoprecipitation (co-IP) assays, 5 × 10^5^ DF-1 cells cultured in 6-well plates were co-transfected with the indicated plasmids co-expressing the full-length M protein and chBRD2 protein or their truncation mutants. At 36 h post-transfection (hpt), cells were washed three times with PBS and then lysed with immunoprecipitation lysis buffer (Pierce, USA). The supernatants were collected after centrifugation and then incubated with an anti-HA or anti-Myc antibody overnight at 4 °C. The immune complexes were recovered by adsorption to protein A + G-Sepharose (Sigma, USA) for 3 h at 4 °C. After three washes with immunoprecipitation lysis buffer, the immunoprecipitates were detected by western blotting using an anti-Myc or anti-HA antibody.

For pull-down assays, the His-chBRD2 fusion protein (4 h of induction with 0.5 mM IPTG at 25 °C) and GST-M fusion protein (4 h of induction with 0.5 mM IPTG at 28 °C) were expressed in *E. coli* BL21 (DE3), and soluble His-chBRD2 and GST-M were purified on Ni–NTA His*Bind Resin (Merck, USA) and Glutathione-Sepharose 4B beads (GE Healthcare, USA), respectively. In the GST pull-down assay, the purified GST-M protein was immobilized on Glutathione-Sepharose 4B beads. After washing with transport buffer, the immobilized protein was incubated with purified His-chBRD2 for 2 h at 4 °C. The beads were then washed three times with transport buffer, and the target protein His-chBRD2 was eluted from the beads and used for SDS-PAGE followed by western blot analysis. In the His pull-down assay, His*Bind Resin-bound His-chBRD2 was incubated with purified GST-M for 2 h at 4 °C. The resins were treated as described above, and the target protein GST-M was detected by western blotting.

### Multiple sequence alignment and computer modelling

The complete coding sequences of the *BRD2* gene from humans and other selected species were retrieved from the GenBank database. The amino acid sequences of the BRD2 protein (huBRD2 as a template) were analysed using the Clustal W multiple alignment algorithm in the MegAlign program of the DNASTAR Lasergene package, version 7.1 (DNASTAR Inc. Madison, WI, USA). The amino acid conservation of the BRD2/ET domain among humans and different species was obtained based on the above multiple sequence alignment results. The three-dimensional crystal structure of the huBRD2/ET domain (Protein Data Bank [PDB] accession no. 6CUI) was used as a template to illustrate the structural difference between the huBRD2/ET domain and chBRD2/ET domain. This modelling was performed with PyMOL software (Schrödinger).

### The effect of the NDV M protein on the transcription of the *chBRD2* gene

To study the effect of the NDV M protein on the transcription of the *chBRD2* gene, 5 × 10^5^ low-passage DF-1 cells grown to 80% confluence in 6-well plates were infected with rSS1GFP at an MOI of 0.01, 0.1 or 1, or transfected with the plasmid pEGFP-M, pEGFP-M(Δ264–313) (M protein deleting residues 264 to 313), or pEGFP-C1 at a dose of 1.0 μg, 2.0 μg or 4.0 μg. The expression levels of the M protein in rSS1GFP-infected DF-1 cells were detected at 6, 12 and 24 hpi, while the expression levels of EGFP-M, EGFP-M(Δ264–313) and EGFP in DF-1 cells transfected with different doses of plasmid were examined at 36 hpt. The relative levels of the selected proteins to those of the control GAPDH were determined by densitometry using ImageJ software version 1.8.0. In addition, quantitative real-time PCR (qRT-PCR) was used to detect the transcription of the endogenous *chBRD2* gene in virus-infected or plasmid-transfected DF-1 cells using a SYBR Premix Ex Taq Kit (Takara Biomedical Technology, China) according to the manufacturer’s instructions. All the reactions were performed in a 10 μL volume containing 5 μL of 2 × SYBR Premix Ex Taq, 200 nM each primer, and 0.2 μL of ROX reference Dye II. The cycling parameters were 1 cycle at 95 °C for 5 s, followed by 40 cycles at 95 °C for 5 s and 60 °C for 31 s. One cycle of melting curve analysis was added for all reactions to verify product specificity. The relative transcription levels of the *chBRD2* gene were normalized to those of the *GAPDH* gene. The threshold cycle 2^−ΔΔCT^ method was used to determine the fold change in gene transcription levels.

### Small interfering RNA (siRNA) treatment and virus infection

The sequences of three pairs of siRNAs designed to knockdown the *chBRD2* gene (GenBank No. XM_015294960) in DF-1 cells are shown in Table 2. Negative control siRNA (Cat. No. 12935-400) and siRNA transfection reagent Lipofectamine™ RNAiMAX were purchased from Thermo Fisher Scientific Company (USA). For transfection with the siRNA against chBRD2, low-passage DF-1 cells grown to 80% confluence in 6-well plates were transfected with the indicated siRNAs (10 μM) at a dose of 30 pmol, and the knockdown efficiency was detected by qRT-PCR at 48 hpt, as described above. To study the effect of chBRD2 knockdown on the replication of NDV, at 48 hpt, rSS1GFP was used to infect chBRD2 siRNA#2- or control siRNA-treated DF-1 cells or non-transfected cells at an MOI of 1. The cell culture supernatants were collected at the indicated time points (6, 12, 24, 36, 48, and 72 hpi), and the virus titres were titrated using 50% tissue culture infective doses (TCID_50_) in DF-1 cells according to the Reed and Muench method [27]. Meanwhile, the cytopathic effect (CPE) in virus-infected cells was observed under an inverted fluorescence microscope, and the expression of the M protein and GFP protein was examined by western blotting at 6, 12 and 24 hpi. The relative levels of the selected proteins compared to control GAPDH expression were determined by densitometry using ImageJ software version 1.8.0.

**Table 2:** Information on chBRD2 siRNAs

Name	siRNA Name	Sequence (5′ → 3′)	
chBRD2 (GenBank no. XM_015294960)	siRNA#1	Sense	CAAACUGCUAUAUCUAUAACA
		Anti-sense	UUAUAGAUAUAGCAGUUUGUG
	siRNA#2	Sense	ACUGCUAUAUCUAUAACAAGC
		Anti-sense	UUGUUAUAGAUAUAGCAGUUU
	siRNA#3	Sense	GCUCCAAGAACUCAAAGAAAG
		Anti-sense	UUCUUUGAGUUCUUGGAGCUG
Control	Negative siRNA	Sense	UUCUCCGAACGUGUCACGUTT
		Anti-sense	ACGUGACACGUUCGGAGAATT

### chBRD2 overexpression and virus infection

To further investigate the effect of chBRD2 overexpression on the replication of NDV, low-passage DF-1 cells at 80% confluence in 6-well plates were transfected with 3.0 μg of pDsRed-chBRD2 or pDsRed-C1 to overexpress DsRed-chBRD2 or DsRed. The transfection efficiency and transcription level of pDsRed-chBRD2 were examined by fluorescence observation and qRT-PCR at 36 hpt, respectively, and then the plasmid-transfected DF-1 cells were infected with rSS1GFGP at an MOI of 1. The cell culture supernatants were collected at the indicated time points (6, 12, 24, 36, 48, and 72 hpi), and the virus titres were determined in DF-1 cells. In addition, the expression levels of the M protein and GFP protein in DsRed-overexpressing cells, DsRed-chBRD2-overexpressing cells and non-transfected cells were examined by western blotting at 6, 12 and 24 hpi. The relative levels of the selected proteins compared to control GAPDH expression were determined by densitometry using ImageJ software version 1.8.0.

### Quantification of viral RNA synthesis and gene transcription by qRT-PCR

The primers used for the quantification of viral RNA synthesis and gene expression by qRT-PCR were designed based on the target sequences using Primer Premier 5.0 software (Table 3). siRNA- or plasmid-transfected DF-1 cells were collected from virus infection assays at the indicated time points (6, 12 and 24 hpi), and total RNA was extracted using TRIzol reagent (Invitrogen, USA) according to the manufacturer’s protocol. One microgram of total RNA per sample was reverse-transcribed using a previously described method [28, 29]. Briefly, the primer PRT-G (5′-ACGATAAAAGGCGGAGAAGCA-3′, nucleotide positions 24–44 in the SS1 genome) specific for negative-sense viral RNA was used for reverse transcription. Reverse transcripts were stored at − 80 °C until use in qRT-PCR assays. The primers qNDV/NP-F and qNDV/NP-R and qNDV/P-F and qNDV/P-R were used to quantify the viral *NP* gene and *P* gene, respectively. On the other hand, 1 μg of total RNA per sample was reverse-transcribed into cDNA using Superscript IV reverse transcriptase (Invitrogen, USA). The primers qNDV/M-F and qNDV/M-R and qGFP-F and qGFP-R were used to quantify the viral *M* gene and *GFP* gene, respectively. qRT-PCR experiments were performed using a SYBR Premix Ex Taq Kit (Takara Biomedical Technology, China). The qRT-PCR procedure used to quantify viral RNA synthesis and gene transcription was carried out according to our previously described method [10]. The relative gene transcription levels were normalized to that of the *GAPDH* gene. The threshold cycle 2^−ΔΔCT^ method was used to determine the fold change in gene transcription levels.

**Table 3:** Quantitative real-time PCR primers used in this study

Primer name	Sequence (5′ → 3′)	Product size (bp)	GenBank no.
qchBRD2-F	AAAGTGGTGATGAAAGCCCTGTGGA	113	XM_015294960
qchBRD2-R	ATGGGCTGCTTGATGATCTTGTGGT		
qNDV/NP-F	TTACAACTTGGTCGGGGATGTAGAC	166	KP742770
qNDV/NP-R	CATCCGATATAAACGCATGAGCTG		
qNDV/P-F	TATGGAAGCAACCAGGGAAGACC	185	KP742770
qNDV/P-R	TGGACACGATCCACAGGTACAGGA		
qNDV/M-F	CTGTGCTTGTGAAGGCGAGAGGT	100	KP742770
qNDV/M-R	TGGGGAGAGGCATTTGCTATAGGAT		
qGFP-F	CGACAAGCAGAAGAACGGCATCA	154	U55763
qGFP-R	GGACTGGGTGCTCAGGTAGTGGTT		
qGAPDH-F	TCAAGGCTGAGAACGGGAAACTTG	117	NM_204305
qGAPDH-R	TGGACTCCACAACATACTCAGCACC		

### Statistical analysis

Differences in the expression levels of genes and proteins and virus titres were analysed using SPSS 12.0 software (SPSS Inc., USA). An independent-samples *t* test was used for data analysis. All experiments were repeated at least three times, and the results are presented as the mean ± standard deviation (SD). A *P*-value < 0.05 was considered significant. P-values are indicated by asterisks (*P < 0.05, **P < 0.01, ***P < 0.001).

## Results

### Interaction of the NDV M protein and chBRD2 protein in cellulo and in vitro

**Figure 1:**
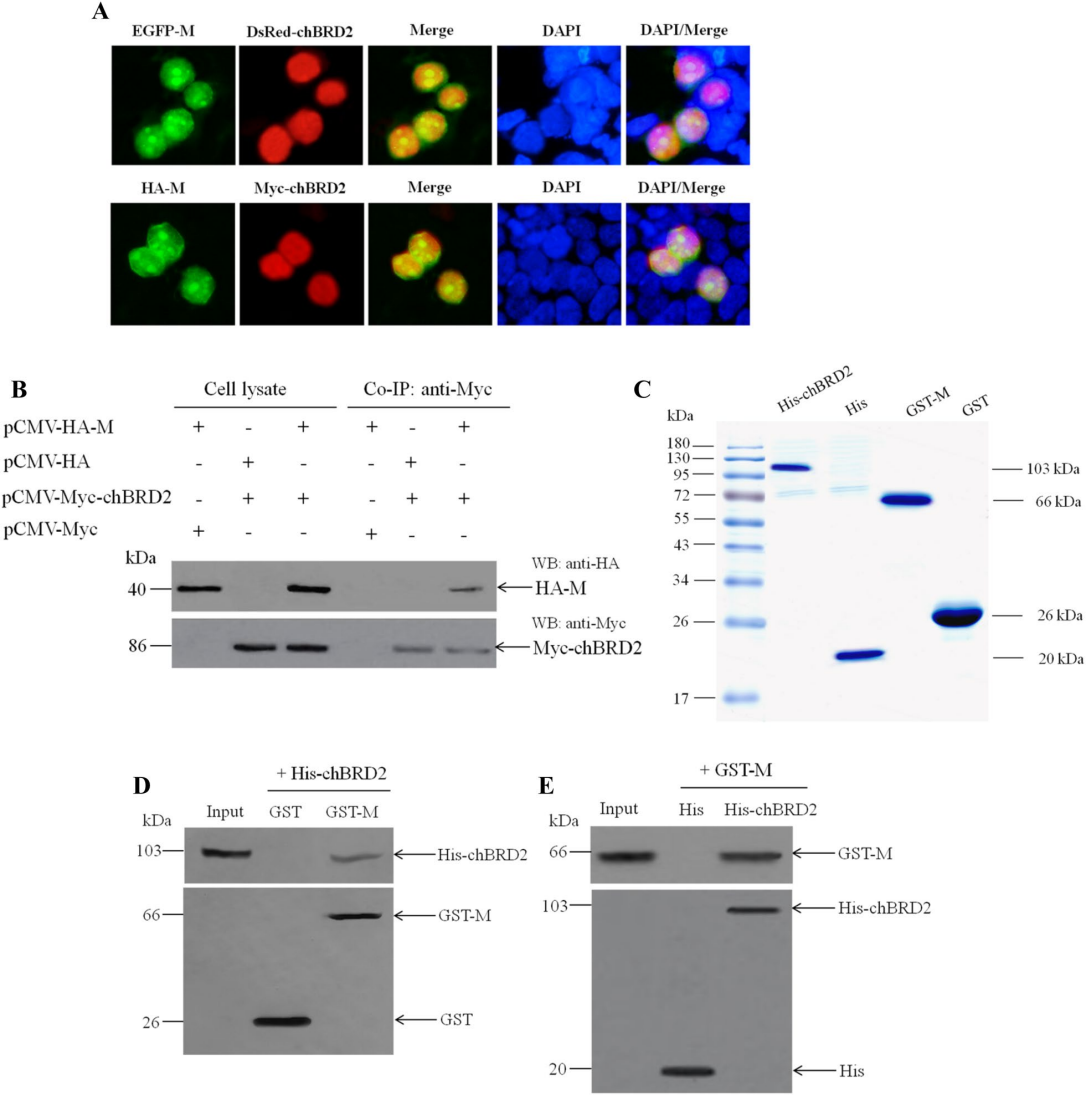
**Identification of the interaction between the NDV M protein and chBRD2 protein. A** Fluorescence co-localization of the NDV M protein and chBRD2 protein in plasmid-co-transfected cells. The plasmids pEGFP-M and pDsRed-chBRD2 or pCMV-HA-M and pCMV-Myc-chBRD2 were co-transfected into DF-1 cells. Twenty-four hours after transfection, direct fluorescence methods and indirect immunofluorescence assays were used to observe the fluorescence of EGFP-M and DsRed-chBRD2 or HA-M and Myc-chBRD2. DAPI was used to detect nuclei. The original magnification was 1 × 200. **B** Identification of the interaction between the M protein and chBRD2 protein by co-IP assay. The indicated plasmids were co-transfected into DF-1 cells, and reciprocal co-IP assays were performed to identify the interaction between HA-M and Myc-chBRD2 in DF-1 cells at 36 hpt. **C** A Coomassie stained gel showing the bacterial expression purified proteins. The His-tagged chBRD2 (His-chBRD2), His tag, GST-tagged M (GST-M), and GST tag were expressed in *E. coli* BL21 (DE3), and the soluble His-chBRD2 and His tag or GST-M and GST tag were purified on Ni–NTA His*Bind Resin or Glutathione-Sepharose 4B beads, respectively. The bacterial expression purified proteins were detected by SDS-PAGE along with Coomassie blue staining. Identification of the interaction between the M protein and chBRD2 protein by His pull-down assay (**D**) and GST pull-down assay (**E**). Upper panel, the purified His-chBRD2 or GST-M (Input) and the pull-downed His-chBRD2 or GST-M were detected by western blot using a mouse anti-His or anti-GST antibody. Lower panel, the bait proteins (GST and GST-M or His and His-chBRD2) in the pull-down fractions were detected by western blot using a mouse anti-GST or anti-His antibody.

The chBRD2 protein is one of the cellular proteins found to interact with the NDV M protein using the yeast two-hybrid screening system [11]. To further confirm their interaction, fluorescence co-localization, co-IP and pull-down assays were performed. As shown in Figure 1A, either EGFP-M and DsRed-chBRD2 fusion proteins or HA-M and Myc-chBRD2 fusion proteins showed clear co-localization in the nucleus in co-transfected DF-1 cells, demonstrating that the nuclear localization of the M protein and chBRD2 protein was not affected by the fused tag. To verify the in cellulo interaction between the M protein and chBRD2 protein, a co-IP assay with DF-1 cells transiently co-transfected with plasmids encoding HA-M and Myc-chBRD2 was carried out. Western blot analysis results showed that the fusion proteins HA-M and Myc-chBRD2 were normally expressed (Figure 1B). Moreover, the HA-M fusion protein but not HA tag in cell supernatants could be immunoprecipitated with Myc-chBRD2 when using an anti-Myc antibody (Figure 1B). In addition, the relatively pure recombinant proteins GST-M and His-chBRD2 were obtained after prokaryotic expression and protein purification (Figure 1C). Moreover, an in vitro binding assay of the fusion protein GST-M to the purified His-chBRD2 fusion protein showed that the His-chBRD2 protein was pulled down by the GST-M protein but not by the GST tag (Figure 1D) and that, in turn, the GST-M protein was also pulled down by the His-chBRD2 protein but not by the His tag (Figure 1E). These results demonstrated that the NDV M protein physically interacted with the chBRD2 protein.

### Mapping the binding domains between the NDV M protein and chBRD2 protein

**Figure 2:**
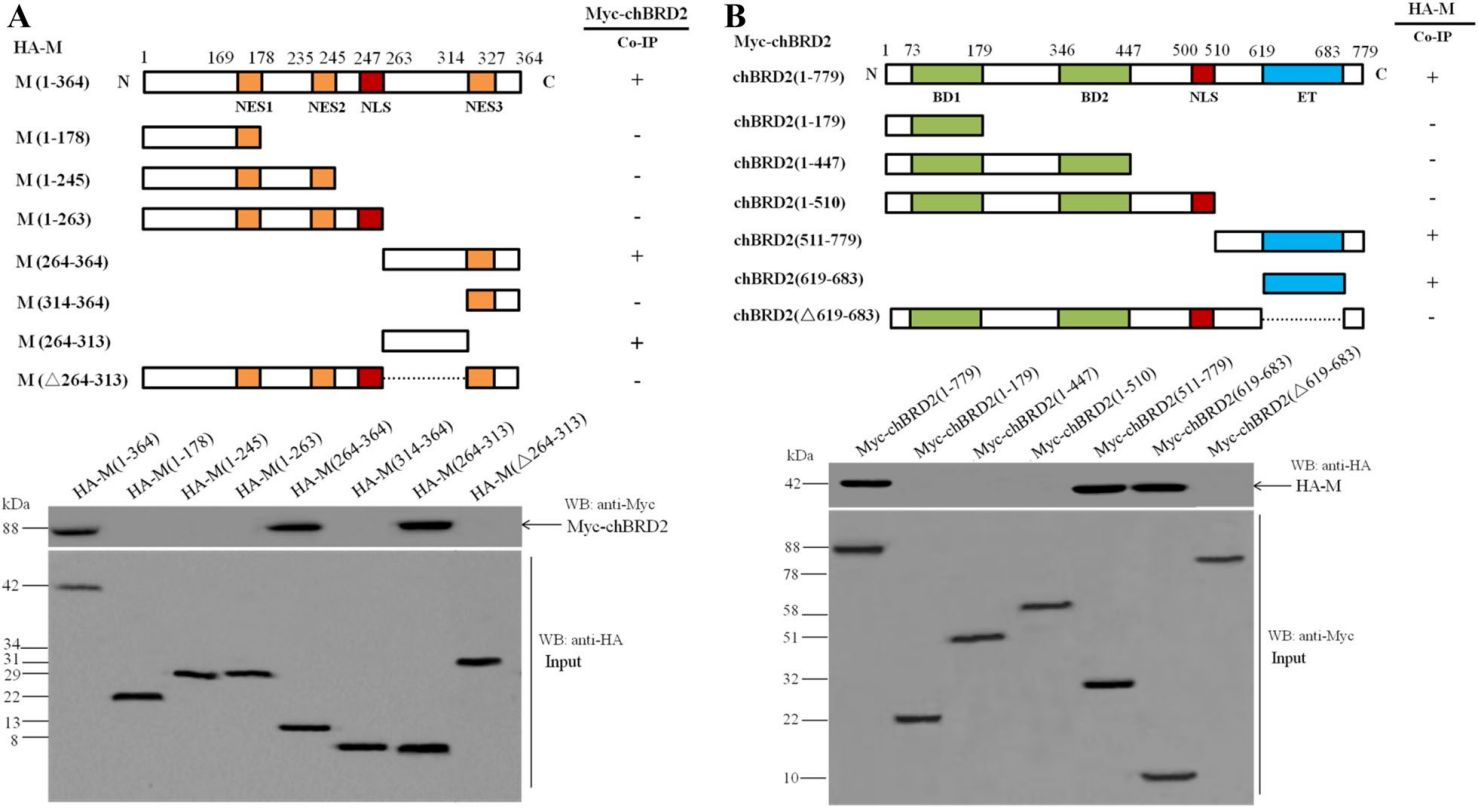
**Characterization of the binding domains between the NDV M protein and chBRD2 protein. A** Mapping the binding domain of the NDV M protein for the chBRD2 protein. Plasmids expressing HA-tagged M or its truncation mutants and the chBRD2 protein were co-transfected into DF-1 cells. Thirty-six hours after transfection, the interactions between HA-tagged M or its truncation mutants and the chBRD2 protein were examined by co-IP assay. Upper panel, schematic representation of the full-length and truncation mutants of the HA-tagged M protein. Lower panel, HA-tagged M or its truncation mutants (Input) interacting with Myc-chBRD2 was detected by western blot. **B** Mapping the binding domain of the chBRD2 protein for the NDV M protein. Upper panel, schematic representation of the full-length and truncation mutants of the Myc-tagged chBRD2 protein. Lower panel, Myc-tagged chBRD2 or its truncation mutants (Input) interacting with HA-M was detected by western blot.

The NDV M protein is reported to contain three NESs and one NLS [12, 30], and the BRD2 protein contains one NLS, two bromodomains (BD1 and BD2), and one extra-terminal (ET) domain [24]. To determine the interaction regions between the M protein and chBRD2 protein, a series of M and chBRD2 truncation mutants were constructed to search for their binding domains using a co-IP assay (Figures 2A and B, upper panels). The binding experiments showed that the C-terminus (aa 264–313) of the M protein was essential for chBRD2 binding since HA-M(1–364), HA-M(264–364) and HA-M(264–313) could be immunoprecipitated with Myc-chBRD2, while the other truncation or deletion mutants of the HA-tagged M protein lost their binding activity to Myc-chBRD2 (Figure 2A, lower panel). In addition, only the fragments containing the ET domain (aa 619–683) of chBRD2, such as Myc-chBRD2(1–779), Myc-chBRD2(511–779) and Myc-chBRD2(619–683), could interact with the HA-M protein (Figure 2B, lower panel). Therefore, these results suggested that the C-terminus (aa 264–313) of the NDV M protein and the ET domain (aa 619–683) of the chBRD2 protein were responsible for interaction with each other.

### Conservation analysis of the BRD2/ET domain among humans and other species

**Figure 3:**
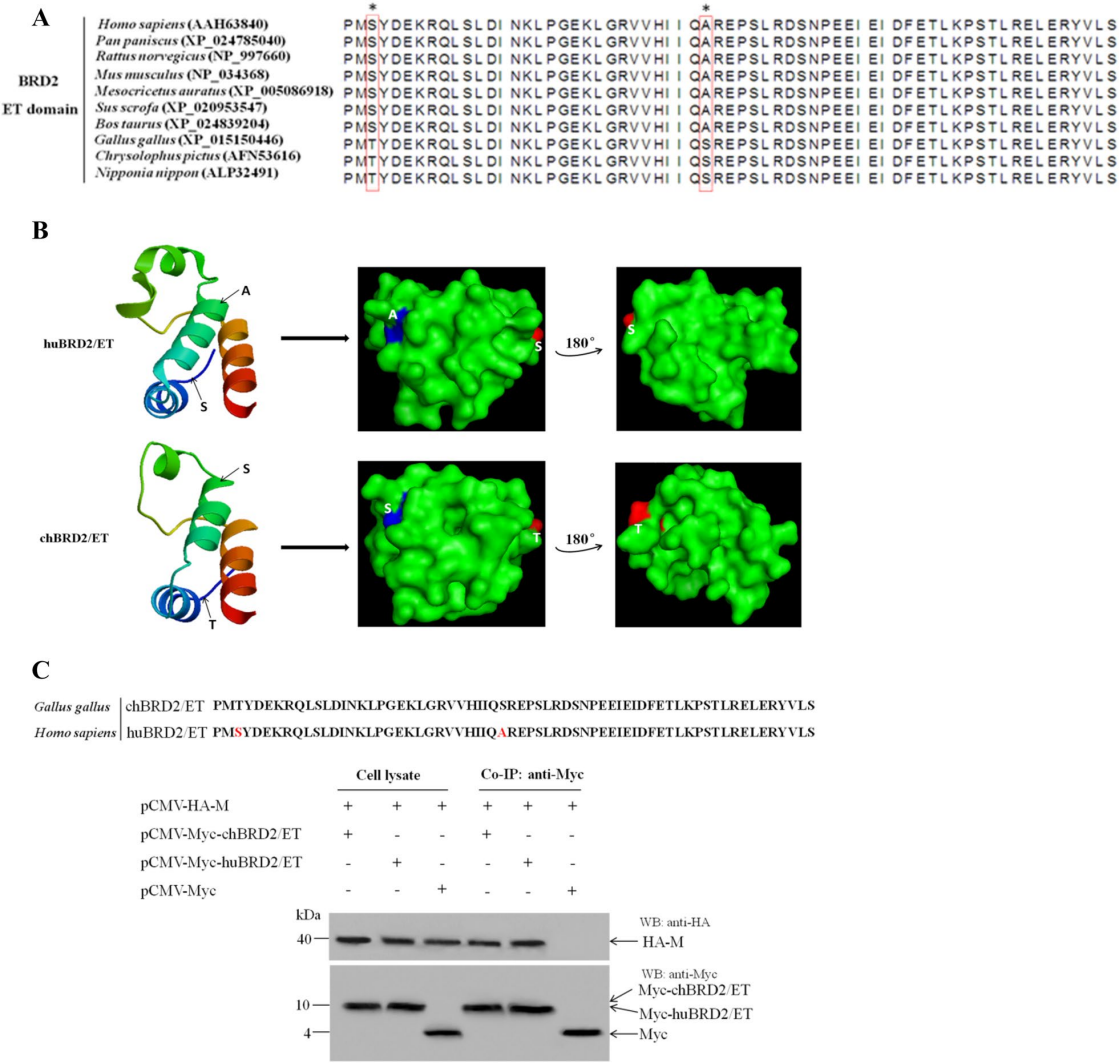
**Conservation analysis of the BRD2/ET domain from different species. A** The complete coding sequences of the *BRD2* gene from humans and other selected species were retrieved from the GenBank database. The amino acid sequences of the BRD2 protein (huBRD2 as the template) were analysed using the Clustal W multiple alignment algorithm in the MegAlign program of the DNASTAR Lasergene package. The different amino acids were marked in boxes and asterisks. **B** The three-dimensional crystal structures of the huBRD2/ET domain and chBRD2/ET domain (PDB accession no. 6CUI) were analysed using PyMOL. The different amino acids, including S in huBRD2/ET and T in chBRD2 and A in huBRD2/ET and S in chBRD2/ET, were marked in red and blue, respectively. **C** Identification of the interaction between the chBRD2/ET or huBRD2/ET domain and NDV M protein. Upper panel, schematic representation of the chBRD2/ET domain and huBRD2/ET domain. Lower panel, the combined plasmids were co-transfected into DF-1 cells, and 36 h after transfection, the interaction between HA-M and Myc-chBRD2/ET or Myc-huBRD2/ET was identified by co-IP assay.

To understand the conservation of the BRD2/ET domain, the protein sequences of BRD2 from avian species (*Gallus gallus*, *Chrysolophus pictus*, and *Nipponia nippon*), humans and other mammals, including *Pan paniscus*, *Rattus norvegicus*, *Mus musculus*, *Mesocricetus auratus*, *Sus scrofa*, and *Bos taurus*, were analysed. The results of multiple sequence alignment showed that the amino acids of the BRD2/ET domain were absolutely conserved between humans and other mammals, but there were two amino acid changes (T621 and S649) found in the chBRD2/ET domain (Figure 3A). Further three-dimensional crystal structure modelling of huBRD2/ET and chBRD2/ET showed that the T621 and S649 changes in the chBRD2/ET domain mainly reduced α-helix formation and obviously changed the molecular structure surface when compared to the huBRD2/ET domain (Figure 3B). However, it is surprising that the two different amino acid changes found in the BRD2/ET domain of humans and other mammals did not disrupt the BRD2-M interaction or the chBRD2-M interaction (Figure 3C). Thus, these data indicated that other regions or sites of the chBRD2/ET domain might contribute to interactions with the NDV M protein.

### The NDV M protein downregulates the transcription of the *chBRD2* gene

**Figure 4:**
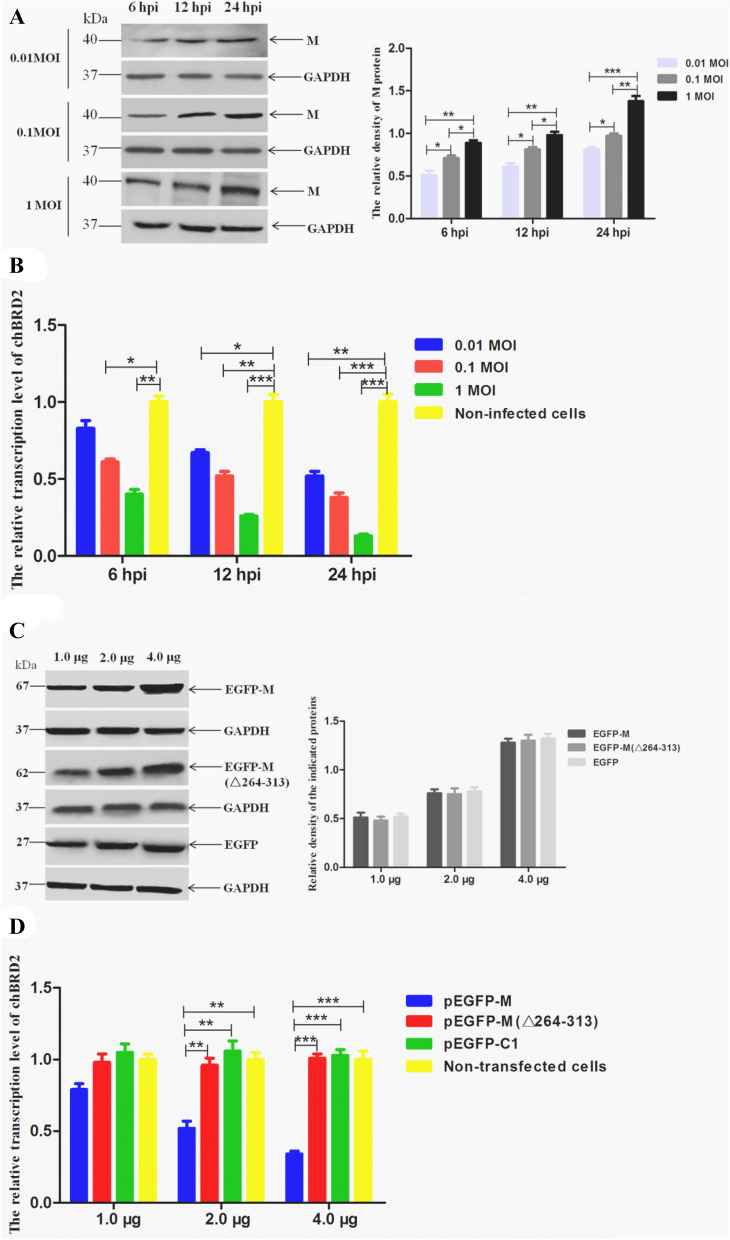
**The effect of the NDV M protein on the transcription of the** ***chBRD2*** **gene. A** The expression levels of the NDV M protein in different infection doses of NDV-infected DF-1 cells. DF-1 cells were infected with rSS1GFP at an MOI of 0.01, 0.1 or 1. The expression levels of the M protein in rSS1GFP-infected DF-1 cells were detected at 6, 12 and 24 hpi. The relative expression levels of the M protein compared to control GAPDH expression levels were determined by densitometry using ImageJ software version 1.8.0. Error bars represent standard deviations (mean ± SD) (*P < 0.05; **P < 0.01; ***P < 0.001 compared to the value of the 0.01 MOI group). **B** Transcription levels of the *chBRD2* gene in DF-1 cells infected with different doses of NDV at 6, 12 and 24 hpi. The relative transcription levels of the *chBRD2* gene were compared with those of the control *GAPDH* gene. Error bars represent standard deviations (mean ± SD) (*P < 0.05; **P < 0.01; ***P < 0.001 compared to the value of non-infected cells). **C** The expression levels of EGFP-M, EGFP-M(△264-313) and EGFP in DF-1 cells transfected with different doses of plasmid. DF-1 cells were transfected with the plasmid pEGFP-M, pEGFP-M(Δ264-313), or pEGFP-C1 at a dose of 1.0 μg, 2.0 μg or 4.0 μg. The expression levels of EGFP-M, EGFP-M(Δ264-313) and EGFP in plasmid-transfected cells were examined at 36 hpt. The relative expression levels of the indicated proteins to control GAPDH expression levels were determined by densitometry using ImageJ software version 1.8.0. **D** Transcription levels of the *chBRD2* gene in DF-1 cells transfected with different doses of pEGFP-M, pEGFP-M(Δ264-313), or pEGFP-C1. The relative transcription levels of the *chBRD2* gene were compared with those of the control *GAPDH* gene. Error bars represent standard deviations (mean ± SD) (**P < 0.01; ***P < 0.001 compared to the value of non-transfected cells).

To determine whether the interaction of the NDV M protein with the chBRD2 protein affects the transcription of chBRD2, the transcription levels of the *chBRD2* gene were evaluated by qRT-PCR in virus-infected or plasmid-transfected DF-1 cells. As shown in Figure 4A, the expression levels of the NDV M protein gradually increased from 0.01 MOI to 1 MOI of NDV strain rSS1GFP at 6, 12 and 24 hpi. In contrast, the transcription levels of the *chBRD2* gene were obviously decreased with increasing infective dose of rSS1GFP from 6 to 24 hpi (downregulated from 0.33 to 0.48 times in the 0.01 MOI group, from 0.39 to 0.62 times in the 0.01 MOI group, from 0.60 to 0.87 times in the 1 MOI group (Figure 4B). Next, different amounts of the plasmid pEGFP-M, pEGFP-M(Δ264–313) or pEGFP-C1 (1.0 μg, 2.0 μg, or 4.0 μg) were transfected into DF-1 cells to examine its effect on the transcription of the *chBRD2* gene. The results showed that the fusion proteins EGFP-M and EGFP-M(Δ264–313) and the EGFP tag more highly expressed when DF-1 cells were transfected with 4.0 μg of plasmid at 48 hpt than after transfection with 1.0 μg or 2.0 μg of each plasmid (Figure 4C). In addition, the transcription levels of the *chBRD2* gene were relatively lower with transfection with 2.0 μg or 4.0 μg of pEGFP-M (downregulated 0.48 times and 0.66 times, respectively) than those of the pEGFP-M(Δ264–313) group, pEGFP-C1 group and non-transfected cells (Figure 4D). Therefore, these results suggested that the NDV M protein could negatively regulate the transcription of the *chBRD2* gene in a dose-dependent manner.

### Knockdown of chBRD2 promotes NDV replication in DF-1 cells

**Figure 5:**
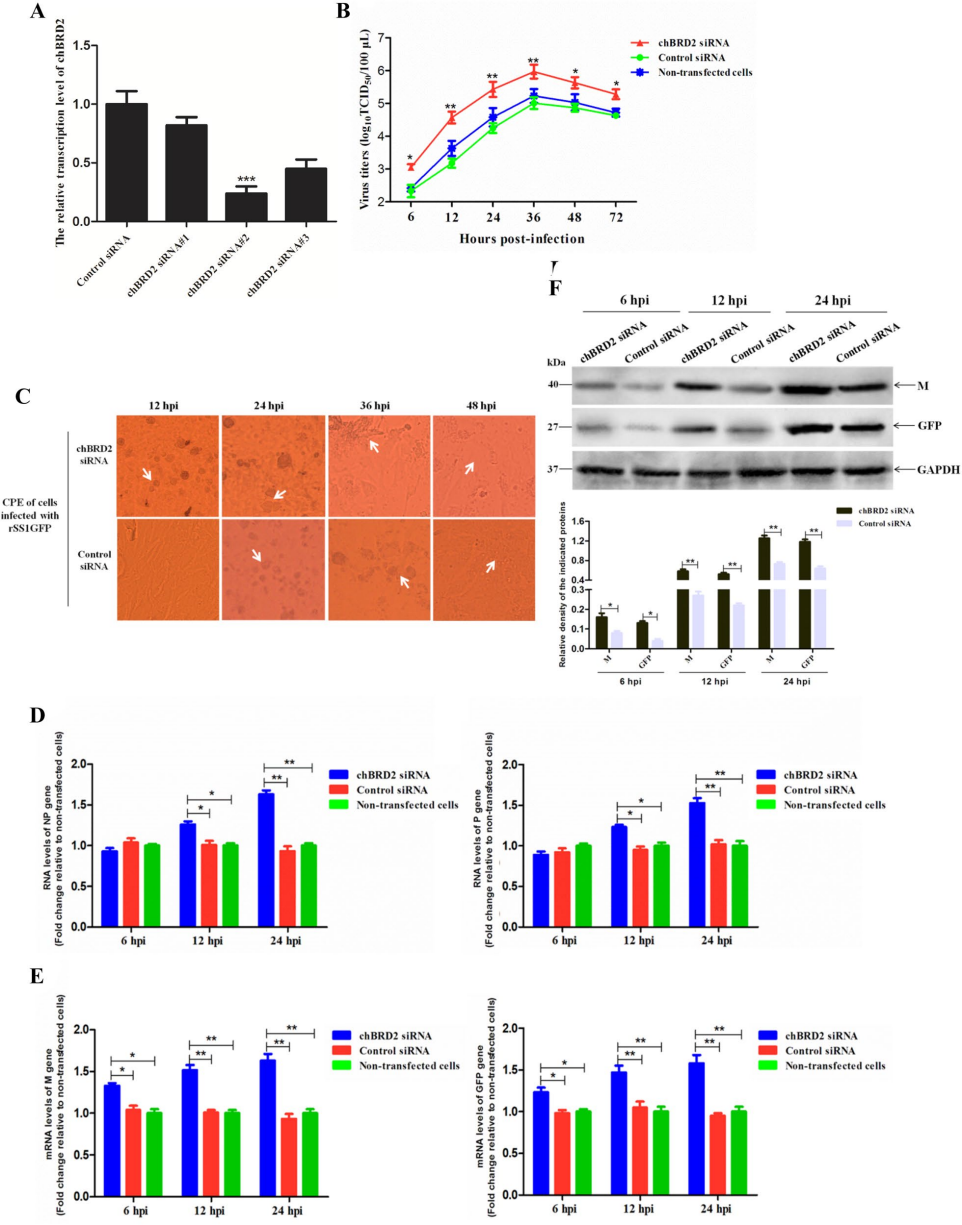
**siRNA-mediated knockdown of chBRD2 promotes NDV replication in DF-1 cells. A** The effect of chBRD2 siRNA or control siRNA on the transcription of the endogenous *chBRD2* gene in DF-1 cells. DF-1 cells grown in 6-well plates were transfected with the indicated siRNAs (10 μM) at a dose of 30 pmol, and the knockdown efficiency of the *chBRD2* gene was detected by qRT-PCR at 48 hpt. The relative transcription levels of the *chBRD2* gene were compared with those of the control *GAPDH* gene. Error bars represent standard deviations (mean ± SD) (***P < 0.001 compared to the value of the control siRNA group). **B** The growth kinetics of rSS1GFP were compared using multicycle growth curves in chBRD2 siRNA#2- or control siRNA-treated cells or non-transfected cells. The cell culture supernatants were collected at the indicated time points (6, 12, 24, 36, 48, and 72 hpi), and the viral titres were titrated using TCID_50_ (50% tissue culture infective dose) in DF-1 cells. Error bars represent standard deviations (mean ± SD) (*P < 0.05; **P < 0.01 compared to the value of non-transfected cells). **C** The CPE of rSS1GFP was observed in chBRD2 siRNA#2- or control siRNA-treated DF-1 cells at 12, 24, 36 and 48 hpi. The original magnification was 1 × 200. Viral RNA synthesis corresponding to the *NP* and *P* genes (**D**) and viral gene transcription corresponding to the *M* and *GFP* genes (**E**) of rSS1GFP in chBRD2 siRNA#2- or control siRNA-treated cells or non-transfected cells was detected by qRT-PCR at 6, 12 and 24 hpi. Error bars represent standard deviations (mean ± SD) (*P < 0.05; **P < 0.01 compared to the value of non-transfected cells). **F** The expression levels of the M and GFP proteins in chBRD2 siRNA#2- or control siRNA-treated cells were examined by western blot at 6, 12 and 24 hpi, respectively. The relative expression levels of the M and GFP proteins compared to control GAPDH expression levels were determined by densitometry using ImageJ software version 1.8.0. Error bars represent standard deviations (mean ± SD) (*P < 0.05; **P < 0.01 compared to the value of the control siRNA group).

To further investigate the role of chBRD2 in the replication of NDV, siRNA-mediated knockdown of chBRD2 in DF-1 cells infected with rSS1GFP was performed. Three pairs of chBRD2 siRNAs (Table 3) were synthesized and then transfected into DF-1 cells. The results of qRT-PCR analysis showed that chBRD2 siRNA#2 could more effectively reduce the transcription level of the *chBRD2* gene than other chBRD2 siRNAs and control siRNA (Figure 5A). The replication ability and cytopathogenicity of rSS1GFP in chBRD2 siRNA#2- or control siRNA-treated DF-1 cells or non-transfected cells were then evaluated. The results of multicycle growth kinetic analysis revealed that the virus titres of rSS1GFP in chBRD2 siRNA#2-treated DF-1 cells were relatively higher (increased from 1.2 to 1.5 titre) than those in control siRNA-treated cells or non-transfected cells from 12 to 36 hpi (*P *< 0.01) (Figure 5B). In addition, the CPE caused by rSS1GFP infection in chBRD2 siRNA#2-treated cells started at 12 hpi, and the cell monolayer began to be destroyed at 36 hpi, but the CPE in control siRNA-treated cells started to appear at 24 hpi, and cell monolayer destruction was not observed at 36 hpi (Figure 5C).

To examine whether the enhanced replication and cytopathogenicity of rSS1GFP was associated with increased viral RNA synthesis and transcription, chBRD2 siRNA#2- or control siRNA-treated cells or non-transfected cells were infected with rSS1GFP, and the viral RNA levels and related gene transcription levels in rSS1GFP-infected cells were analysed by qRT-PCR. The results showed that the relative viral RNA levels (corresponding to the *NP* and *P* genes) between chBRD2 siRNA#2- and control siRNA-treated cells or non-transfected cells had statistically significant differences at 12 hpi (*P *< 0.05), which continued to increase at 24 hpi (*P *< 0.01) (Figure 5D). In addition, the relative viral gene transcription levels (corresponding to the *M* and *GFP* genes) in chBRD2 siRNA#2-treated cells were more increased than those in control siRNA-treated cells or non-transfected cells at 6 hpi (*P *< 0.05) and significantly higher at 12 and 24 hpi (*P *< 0.01) (Figure 5E). Consistent with the relative transcription levels of the *M* and *GFP* genes, the expression levels of the M and GFP proteins were also increased more in chBRD2 siRNA#2-treated cells than in control siRNA-treated cells at different time points (Figure 5F). Together, these results indicated that knockdown of chBRD2 promoted the replication of NDV by upregulating viral RNA synthesis and transcription at early time points after infection.

### Overexpression of chBRD2 restricts NDV replication in DF-1 cells

**Figure 6:**
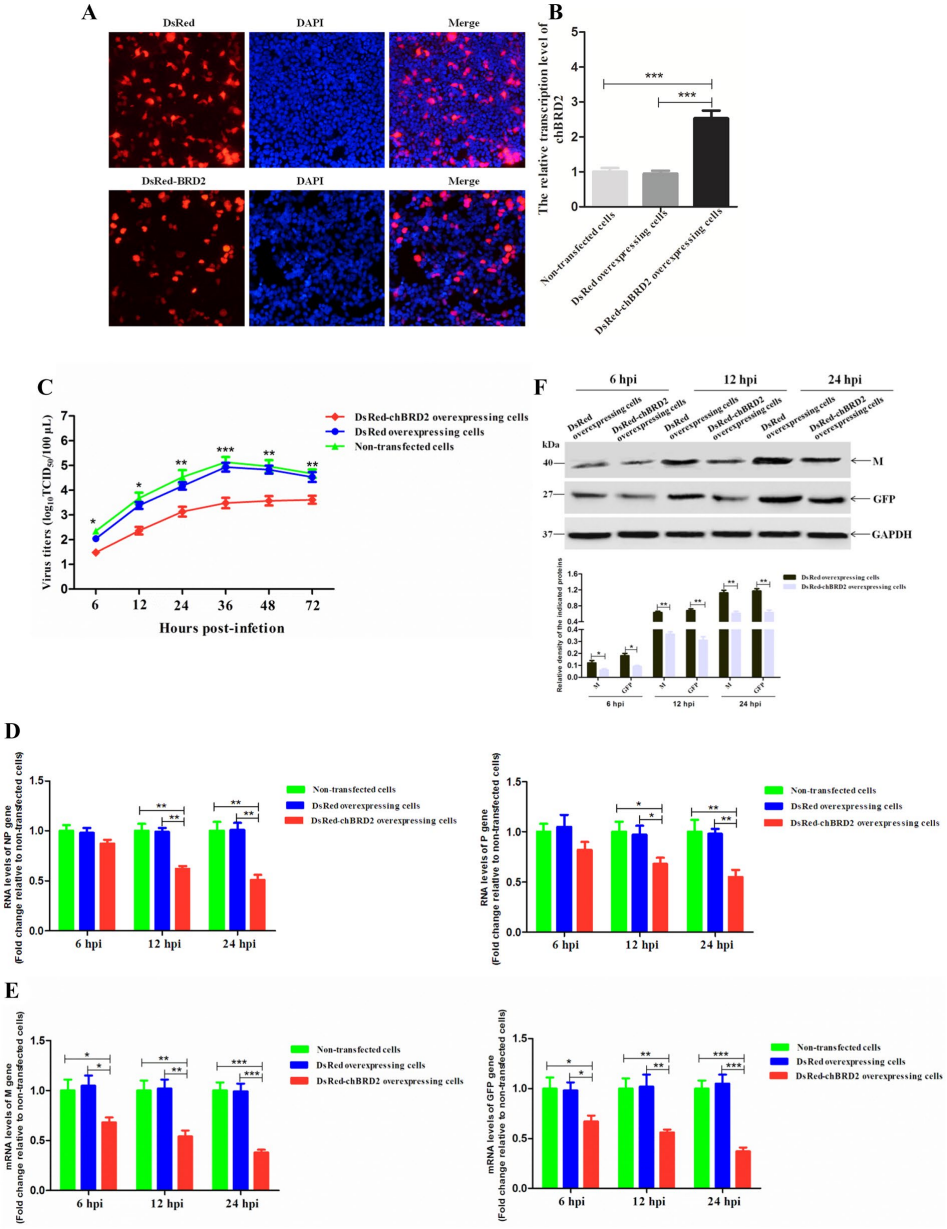
**Overexpression of chBRD2 reduces NDV replication in DF-1 cells. A** Fluorescence observation of DsRed and DsRed-BRD2 in plasmid-transfected DF-1 cells. DF-1 cells were transfected with 3.0 μg of pDsRed-C1 or pDsRed-chBRD2 to overexpress DsRed or DsRed-chBRD2. The fluorescence of DsRed and DsRed-chBRD2 was observed under an inverted fluorescence microscope at 36 hpt. DAPI was used to detect nuclei. The original magnification was 1 × 100. **B** The effect of DsRed or DsRed-BRD2 overexpression on the transcription of the *chBRD2* gene in DF-1 cells. The transcription levels of the *chBRD2* gene in pDsRed-C1- or pDsRed-chBRD2-transfected cells or non-transfected cells were detected by qRT-PCR at 36 hpt. The relative transcription levels of the *chBRD2* gene were compared with those of the control *GAPDH* gene. Error bars represent standard deviations (mean ± SD) (***P < 0.001 compared to the value of non-transfected cells). **C** The growth kinetics of rSS1GFP were compared using multicycle growth curves in pDsRed-C1- or pDsRed-chBRD2-transfected cells or non-transfected cells. The cell culture supernatants were collected at the indicated time points (6, 12, 24, 36, 48, and 72 hpi), and the viral titres were titrated using TCID_50_ (50% tissue culture infective doses) in DF-1 cells. Error bars represent standard deviations (mean ± SD) (*P < 0.05; **P < 0.01; ***P < 0.001 compared to the value of non-transfected cells). Viral RNA synthesis corresponding to the *NP* and *P* genes (**D**) and viral gene transcription corresponding to the *M* and *GFP* genes (**E**) of rSS1GFP in pDsRed-C1- or pDsRed-chBRD2-transfected cells or non-transfected cells was detected by qRT-PCR at 6, 12 and 24 hpi. Error bars represent standard deviations (mean ± SD) (*P < 0.05; **P < 0.01; ***P < 0.001 compared to the value of non-transfected cells). **F** The expression levels of the M and GFP proteins in pDsRed-C1- or pDsRed-chBRD2-transfected cells or non-transfected cells were examined by western blot at 6, 12 and 24 hpi, respectively. The relative expression levels of the M and GFP proteins compared to control GAPDH expression levels were determined by densitometry using ImageJ software version 1.8.0. Error bars represent standard deviations (mean ± SD) (*P < 0.05; **P < 0.01 compared to the value of non-transfected cells).

Because chBRD2 knockdown affected NDV replication, it was intriguing to further determine whether chBRD2 overexpression could also affect NDV replication. Therefore, DF-1 cells overexpressing DsRed or DsRed-chBRD2 were generated. The expression of DsRed or DsRed-chBRD2 in DF-1 cells was obviously observed by fluorescence observation (Figure 6A). The results of qRT-PCR analysis showed that the transcription level of the *chBRD2* gene in pDsRed-chBRD2-transfected cells was much higher than that in pDsRed-C1-transfected cells or non-transfected cells (*P *< 0.001) (Figure 6B). In addition, overexpression of DsRed-chBRD2 reduced the virus titres (from 0.5 to 1.1 titre) of rSS1GFP from 24 to 72 hpi, which especially decreased the virus titres at 36 hpi (*P *< 0.001) (Figure 6C). Then, the effect of chBRD2 overexpression on viral RNA synthesis and transcription was investigated. As shown in Figure 6D, the relative viral RNA levels (corresponding to the *NP* and *P* genes) in DsRed-chBRD2-overexpressing cells were more decreased at 12 and 24 hpi than those in DsRed-overexpressing cells or non-transfected cells. In addition, the relative transcription levels of the *M* and *GFP* genes in DsRed-chBRD2-overexpressing cells were much lower than those in DsRed-overexpressing cells or non-transfected cells at 6 hpi (*P *< 0.05) and especially decreased (from 0.45 to 0.56 times) at 12 and 24 hpi (*P *< 0.01) (Figure 6E). Meanwhile, the transcription expression levels of the M and GFP proteins were also obviously decreased (from 0.49 to 0.58 times) in DsRed-chBRD2-overexpressing cells in comparison to those in DsRed-overexpressing cells at 12 and 24 hpi (Figure 6F). Overall, these results suggested that overexpression of chBRD2 could reduce the replication of NDV by downregulating viral RNA synthesis and transcription.

## Discussion

Studying virus-host protein–protein interactions is an important strategy to better understand the functions of viral proteins [31, 32]. Regarding paramyxovirus M protein and host protein interactions, it has been reported that the interaction of the Nipah and Hendra virus M proteins with AP3B1 protein promotes virus-like particle (VLP) production [33]. In addition, the angiomotin-like 1 protein interacts with the parainfluenza virus 5 (PIV5) M protein [34] and acts as a linker between the PIV5 M and NEDD4 ubiquitin ligases [35], which reveals a novel host factor recruitment strategy for paramyxoviruses to achieve VLP production and virus budding. Moreover, a recent study showed that annexin A2 interacting with the measles virus (MeV) M protein mediates the plasma membrane localization of the M protein and assists the assembly and budding of progeny virions [36]. All of these findings accelerate our understanding of the potential functions of paramyxovirus M proteins. For the NDV M protein, one familiar function is the core role of the M protein in the assembly and budding of NDV [7, 13], and another function is the hypothesized transcriptional inhibition of the M protein in the nucleus, which is analogous to the M proteins of human respiratory syncytial virus (HRSV) [37], vesicular stomatitis virus (VSV) [38] and MeV [39]. In recent years, although several studies have demonstrated that the interactions of the M protein with host proteins are crucial for the replication and pathogenicity of NDV [11, 18–20], none of these host proteins are associated with nuclear proteins or the budding functions of the NDV M protein. Therefore, additional NDV M-interacting host proteins remain to be further identified and studied.

In our previous study, the chBRD2 protein was identified as a new NDV M-interacting partner through yeast two-hybrid screening [11]. BRD2 is known as a nuclear-localized serine-threonine kinase and plays pivotal roles in the transcriptional control of diverse genes [21, 23]. However, the role of the NDV M-chBRD2 interaction in the replication of NDV remains unclear. In this study, the interaction between the M protein and chBRD2 protein was first confirmed by fluorescence co-localization, co-IP and pull-down assays (Figure 1). In addition, we found that the C-terminus (aa 264–313) of the M protein and the ET domain (aa 619–683) of the chBRD2 protein were the binding domains (Figure 2). It has been shown that chBRD2 mainly contains two tandem BD domains and an ET domain. The BD domain is found to bind acetyl-lysine residues in histones and is required for the epigenetic regulation of gene transcription by interacting with nucleosomes within chromatin [24, 25], while the ET domain mainly performs its transcriptional regulatory function by recruiting specific effector proteins [26]. To date, viral proteins reported to interact with the BRD2/ET domain are the latency-associated nuclear antigen (LANA) of Kaposi sarcoma-associated herpesvirus (KSHV) [40] and the integrases of murine leukaemia virus (MLV) [41, 42] and porcine endogenous retrovirus [43]. Moreover, studies also found that amino acids, including R648, S651 and E685/E689, on the surface of the globular huBRD2/ET domain are responsible for the interaction with KSHV LANA [40]. Here, our findings showed that although two amino acids found in the chBRD2/ET domain were not similar to those in the BRD2/ET domain of humans and other mammals and changed the molecular structure surface of the chBRD2/ET domain (Figures 3A, B), they did not disrupt the BRD2-M interaction or the chBRD2-M interaction (Figure 3C). It is important that the above amino acids (R648, S651 and E685/E689) in the globular huBRD2/ET domain are also conserved in avian species and other mammals (Figure 3A). Therefore, other regions or sites of the chBRD2/ET domain might be responsible for its interaction with the NDV M protein.

It has been demonstrated that M nuclear localization in some cytoplasmic RNA viruses, such as MeV and VSV, can inhibit host cell transcription independently of other viral components. For example, transiently overexpressed MeV M protein in plasmid-transfected cells binds to nuclear factors and inhibits in vitro transcription in a dose-dependent manner [39]. Other studies reported that the VSV M protein directly inhibits host cell transcription by inactivating host RNA polymerases I and II [44] and interacts with nuclear pore complexes to impair nuclear export of cellular mRNAs, which indirectly leads to a decrease and an increase in host cell and virus gene transcription, respectively [45–47]. Recently, we revealed that nuclear localization of the NDV M protein possibly affected host cell transcription because transcription repressor activity-related differentially expressed (DE) genes were upregulated and transcriptional activator activity-related DE genes were downregulated [10], and the expression of most DE proteins related to “transcription” and “RNA processing and modification” was significantly decreased in NDV-infected cells early in infection when compared to that in cells infected with the mutant NDV harbouring M/NLS mutation [48]. More importantly, these upregulated or downregulated DE genes participated in regulating viral RNA synthesis and NDV replication [10]. In this study, we found that the transcription levels of the *chBRD2* gene were obviously decreased in both virus-infected cells and pEGFP-M-transfected cells in a dose-dependent manner (Figure 4). Meanwhile, siRNA-mediated knockdown of chBRD2 promoted NDV replication by upregulating viral RNA synthesis and transcription (Figure 5), while overexpression of chBRD2 produced the opposite results in comparison to those of the chBRD2 knockdown experiment (Figure 6). Together, these findings suggested that downregulation of cellular transcription-related genes caused by M nuclear localization was beneficial for viral RNA synthesis and transcription to promote NDV replication.

BRD2 is a TATA-binding protein (TBP)-associated protein that recruits TBP into the E2F-1 transcriptional complex to perform transactivation effects [26]. In addition, a previous study showed that the major interaction subcomplexes of BRD2 include TBP-associated factors (TAFs), RNA Polymerase II, activated transcription factors, chromatin/histone modification enzymes and SWI/SNF remodelling complex components [49]. However, whether the NDV M-chBRD2 interaction reduces the expression of chBRD2-interacting proteins or chBRD2-involved transcription complexes and how this interaction regulates viral RNA synthesis and transcription must be determined. It is worth noting that the ET domains of BRD2, BRD3 and BRD4 are also very well conserved among human, mammalian and avian species [21, 24]. Thus, the chBRD3 and chBRD4 ET domains might also interact with the NDV M protein. In addition, some studies have reported that the interactions of the BRD2, BRD3, and BRD4 ET domains with MLV integrase stimulate its catalytic activity and promote efficient MLV integration near active transcription units in the host genome [41, 50]. Additionally, the ET domains of BRD2 and BRD4 binding on the surface of KSHV LANA (kLANA) are required for the formation of kLANA “nuclear speckles” and latent replication [40]. Unlike the functions of the BRD2/ET domain interacting with KSHV LANA and MLV integrase, NDV undergoes genomic replication, mRNA transcription, protein synthesis, and assembly of virus components in the cytoplasm, but the NDV M-chBRD2/ET interaction in the nucleus mainly assists in viral RNA synthesis and transcription until later in infection. Currently, accumulating studies have also revealed that the BRD2, BRD3 and BRD4 proteins participate in the host immune response against virus infection [51–53]. In our recent studies using quantitative proteomics analysis, we found that the parental virus rSS1GFP but not the mutant virus rSS1GFP-M/NLSm harbouring an NLS mutation in the M protein reduced the expression of the BRD2, BRD3 and BRD4 proteins to different degrees in virus-infected cells, suggesting that the decreased expression of BRD2, BRD3 and BRD4 was caused by early nuclear localization of the M protein [48]. Here, we also found that chBRD2 knockdown or overexpression continued to increase or reduce the viral titres in DF-1 cells, respectively, indicating that chBRD2 might be involved in the immune response against NDV infection. Therefore, further studies will be performed to identify the interactions between the M protein and the chBRD3 and chBRD4 ET domains and to elucidate the role of chBRD2, chBRD3 and chBRD4 in the regulation of NDV replication in detail.

In conclusion, we demonstrated in this study that the NDV M protein interacted with the chBRD2 protein and downregulated its expression to facilitate viral RNA synthesis and transcription, which was beneficial for NDV replication. These findings will provide valuable information for better understanding the potential biological functions of M’s nuclear localization in the NDV life cycle and aid in our understanding of the molecular mechanisms of NDV replication.

## Data Availability

The datasets used and/or analysed during the current study are available from the corresponding author on reasonable request.

## Abbreviations

BD: bromodomain
BET: bromodomain and extra-terminal domain
BRD2: bromodomain-containing protein 2
BRD3: bromodomain-containing protein 3
BRD4: bromodomain-containing protein 4
co-IP: co-immunoprecipitation
CPE: cytopathic effect
ET: extra-terminal
hpi: hours post-infection
hpt: hours post-transfection
HRSV: human respiratory syncytial virus
KSHV: Kaposi sarcoma-associated herpesvirus
LANA: latency-associated nuclear antigen
MeV: measles virus
MLV: murine leukaemia virus
NDV: Newcastle disease virus
NES: nuclear export signal
NLS: nuclear localization signal
qRT-PCR: quantitative real-time PCR
siRNA: small interference RNA
TCID_50_: 50% tissue culture infective doses
VLP: virus-like particle
VSV: vesicular stomatitis virus
